# Torus Mandibularis in Patients Receiving Hemodialysis

**DOI:** 10.3390/ijerph18189451

**Published:** 2021-09-07

**Authors:** Pei-Ching Chang, Shao-Yu Tai, Chia-Lin Hsu, Aileen I. Tsai, Jen-Fen Fu, I-Kuan Wang, Cheng-Hao Weng, Tzung-Hai Yen

**Affiliations:** 1 Department of Pediatric Dentistry, Chang Gung Memorial Hospital, Taoyuan Branch, Taoyuan 333, Taiwan; pearl.pcchang@gmail.com; 2 Department of Pediatric Dentistry, Chang Gung Memorial Hospital, Linkou Branch, Taoyuan 333, Taiwan; jenny3350528@cgmh.org.tw (S.-Y.T.); mr7482@cgmh.org.tw (C.-L.H.); ait001@adm.cgmh.org.tw (A.I.T.); 3 Department of Medical Research, Chang Gung Memorial Hospital, Linkou Branch, Taoyuan 333, Taiwan; cgfujf@cgmh.org.tw; 4 College of Medicine, Chang Gung University, Taoyuan 333, Taiwan; drweng@seed.net.tw; 5 Department of Nephrology, China Medical University Hospital, Taichung 404, Taiwan; ikwang@mail.cmuh.org.tw; 6 College of Medicine, China Medical University, Taichung 406, Taiwan; 7 Department of Nephrology, Clinical Poison Center, Chang Gung Memorial Hospital, Linkou Branch, Taoyuan 333, Taiwan

**Keywords:** torus mandibularis, oral torus, hemodialysis, hyperparathyroidism, parathyroid hormone, osteodystrophy, age, blood phosphate level

## Abstract

Reports on the prevalence of torus mandibularis among dialysis patients have been limited and inconclusive. A wide variety of oral manifestations has been found in patients with hyperparathyroidism. Furthermore, uremia-related changes in facial bone structures have been described in the literature. This prospective observational study examined 322 hemodialysis patients treated at the Chang Gung Memorial Hospital from 1 August to 31 December 2016. Two subgroups were identified: patients with torus mandibularis (n = 25) and those without (n = 297). Clinical oral examinations including inspection and palpation were employed. Our study found that most mandibular tori were symmetric (84.0%), nodular (96.0%), less than 2 cm in size (96.0%), and located in the premolar area (92.0%). Poor oral hygiene was observed among these patients, with 49.7% and 24.5% scoring 3 and 4, respectively, on the Quigley-Hein plaque index. More than half (55.0%) of patients lost their first molars. Multivariate logistic regression analysis revealed that blood phosphate level (odds ratio = 1.494, *p* = 0.029) and younger age (odds ratio = 0.954, *p* = 0.009) correlated significantly with torus mandibularis. The prevalence of torus mandibularis in patients receiving hemodialysis in this study was 7.8%. Younger age and a higher blood phosphate level were predictors for torus mandibularis in these patients.

## 1. Introduction

Torus mandibularis is a benign developmental anomaly comprising condensed cortical bone with a restricted volume of bone marrow [1]. The threshold level of certain genetic and environmental factors being crossed is believed to be the trigger for torus mandibularis [2,3]. Studies have found that local factors play a role in the formation of these tori, such as parafunctional activity [2], temporomandibular disorder [4], and bone mineral density [5].

Torus mandibularis is rare among juveniles. Its prevalence increases after childhood and plateaus during adulthood [6]. Factors such as increased muscle strength, masticatory stress, and number of existing teeth appear to be associated with the presence of mandibular tori [7]. These tori may develop due to the forces exerted upon cusps and those from the palatine process to the lingual side of alveolar process. Hence, the occurrence of tori appears to be a compensatory and protective bone reaction to occlusal overload in the most vulnerable area of the mandible [8]. An increase in the size of torus mandibularis [9] may inform the clinical assessment of occlusal stress [10]. As the force per unit area is reduced from more occlusal contact points, the wear on teeth also decreases over time.

Reports on the prevalence of torus mandibularis among dialysis patients remain limited and inconclusive. A wide variety of oral manifestations has been found in patients with hyperparathyroidism [11]. In patients receiving dialysis, biochemical disturbances may trigger augmented synthesis of parathyroid hormone and secondary hyperparathyroidism, which in turn produces the bony disorders specific to kidney osteodystrophy [12]. Torus mandibularis could thus be the consequence of biomechanical forces, which reduce the cortical bone and enlarge the trabecular bone. Padbury et al. [13] reported that patients suffering primary hyperparathyroidism were likely to develop oral tori and experience diminutions in radicular lamina dura. Rai et al. [14] also found a higher circulating level of parathyroid hormone in patients with primary hyperparathyroidism in additional to frequent occurrences of loss of lamina dura, ground-glass finding, and a lessening in the mandibular cortical width in these patients. Furthermore, uremia-related changes in facial bone structures have been stated in literature [15,16,17]. For example, Bakathir et al. [15] described the progressive enlargement of facial bones of a 21-year-old female patient with uremia whose facial enlargement involved the maxilla and caused facial and dental deformities. Lopes et al. [16] reported two uremic females with facial disfigurement affecting the maxilla and the mandible. Raubenheimer et al. [17] also documented two female uremic cases with extensive jaw lesions due to secondary hyperparathyrodism. In our previous study on torus mandibularis, a prevalence rate of 6.75% was found among 119 hemodialysis patients [18]. Given the small sample size of this earlier study, the objective of the current study was to examine the prevalence of torus mandibularis among a larger group of hemodialysis patients. In addition, our study aimed to explore the impact of potential pathogenic factors, such as the mineral and bone disorder associated with chronic kidney disease, hyperparathyroidism, nutrition, and age, on the formation of mandibular tori.

## 2. Materials and Methods

### 2.1. Sample Size

There are few discussions in the literature about the risk factors for torus mandibularis in hemodialysis patients. Previous research [19] has also found a significantly higher apnea-hypopnea index in this patient population (*p* = 0.006) in. Hence, a decision was made to determine the sample size for our study using the apnea-hypopnea index. The G*Power software (version 3.1.9.7, https://stats.idre.ucla.edu/stata/ado/analysis/, accessed on 31 July 2021) was employed and a sample size of 78 was derived (effect size = 0.384; alpha = 0.05; power = 0.95).

### 2.2. Inclusion and Exclusion Criteria

Recruitment was conducted among hemodialysis patients who were treated at the hemodialysis center of the Chang Gung Memorial Hospital from 1 August to 31 December 2016. Any patient who signed the informed consent form was eligible to participate. A total of 322 hemodialysis patients signed on and was included in this prospective observational study. Exclusion criteria included cancer, hospitalization (at the time of the study), any operation 3 months prior to the study, and a history of hemodialysis shorter than 6 months. These exclusion criteria were administered to eliminate potential confounding factors related to serum biochemistry and electrolytes which might accompany these events.

### 2.3. Patient Groups

Patients enrolled in the current study were classified into one of the following subgroups: those with torus mandibularis (n = 25) and those without (n = 297).

### 2.4. Hemodialysis Prescription

All patients were on maintenance hemodialysis, 3 times a week for 4 h each time. Hemodialysis was performed with single-use hollow-fiber artificial kidneys fitted with modified cellulose, polyamide, or polysulfone membranes. The dialysate was a standard ionic composition with bicarbonate-based buffer. A standard reverse osmosis system was employed for water purification.

### 2.5. Clinical Oral Examinations

Mandibular tori were examined using mouth mirrors and flashlight through clinical inspection and palpation. Any unobservable thickening or roughness which could be identified only through palpation was considered a normal lingual contour. Once a torus was detected, its location, size, and clinical characteristics were recorded. The size of the torus was determined by measuring the maximum lingual projection or thickness of the torus with a periodontal probe, and noted as ≥2 cm or <2 cm [20]. The shapes of tori were categorized as flat, spindle, nodular, or lobular [21]. The locations of tori were labeled as in one or more of the following areas: incisor, premolar, and molar. The molar relationship was graded as none, Class I, Class II, or Class III, according to the Angle’s classification of malocclusion [22]. For participants’ oral hygiene, the plague on the buccal and lingual nonrestored surfaces of teeth was rated—on a scale of 0 to 5—based on the Turesky-Gilmore-Glickman modification of the Quigley-Hein plaque index (TQHPI) [23].

### 2.6. Statistical Analysis

Continuous variables were described using means and standard deviations, and categorical variables were described using numbers with percentages of the total in parentheses. The Student’s *t*-test (for continuous variables) and chi-square tests (for categorical variables) were conducted for comparisons between patients with torus mandibularis and those without. Univariate binary logistic regression analysis was performed to analyze potential variables that might be associated with the formation of torus mandibularis. To control for confounders, stepwise backward multivariate binary logistic regression was carried out to analyze the variables which were found to be significant from the univariate analysis. The statistical significance was set at 95% confidence interval. All analyses were performed using IBM SPSS Statistics Version 20.0 (IBM, Armonk, NY, USA).

## 3. Results

The average age in years of participating patients was 61.1 ± 11.8 and they had been receiving dialysis for 107.1 ± 82.4 months. Hypertension, diabetes mellitus, and bruxism were found in 50.9%, 35.4%, and 10.2% of patients, respectively. Patients with torus mandibularis were younger than those without (54.4 ± 10.4 versus 61.6 ± 11.8, *p* = 0.003). There were no significant statistical differences in other demographics, health conditions, and lifestyle factors between the two groups (Table 1).

Blood sampling was routinely performed in patients receiving chronic hemodialysis for the following (with markers in parentheses): biochemistry and electrolytes (fasting glucose, sodium, potassium, chloride, calcium, phosphorus, uric acid, and bicarbonate), renal function (blood urea nitrogen, creatinine, and estimated glomerular filtration rate), liver function (aspartate aminotransferase, alanine aminotransferase, gamma-glutamyl transferase, and alkaline phosphatase), lipid profile (total cholesterol, high-density lipoprotein, low-density lipoprotein, and triglyceride), hemogram (white blood cell count, red blood cell count, hemoglobin, hematocrit, mean corpuscular volume, mean corpuscular hemoglobin, mean corpuscular hemoglobin concentration, red blood cell distribution width, and platelet count), ferrokinetics (iron, total iron binding capacity, and ferritin), parathyroid hormone (intact parathyroid hormone), nutrition (albumin), and inflammation (high-sensitivity C-reactive protein). As presented in Table 2, patients with torus mandibularis had a higher blood phosphate level than patients without torus mandibularis (5.6 ± 1.3 versus 5.0 ± 1.1 mg/dL, *p* = 0.010). Patients with torus mandibularis also had a lower blood intact parathyroid hormone level, although the difference between the two groups did not reach statistical significance (297.3 ± 232.0 versus 328.1 ± 317.3 pg/mL, *p* = 0.636). The two patient groups did not differ in inflammatory markers such as high-sensitivity C-reactive protein (8.1 ± 10.3 and 6.8 ± 11.1 mg/L, *p* = 0.582) or nutrition markers such as albumin (3.9 ± 0.3 and 4.0 ± 0.3 g/dL, *p* = 0.656). No significant differences in other laboratory data were noted.

Table 3 presents the dialysis-related data for patients with tori and those without. The Kt/V, urea reduction ratio, and time-averaged concentration of urea are markers of hemodialysis adequacy, and normalized protein catabolic rate is a marker of nutrition. There was no difference in these readings between patients with tori and those without.

The prevalence of torus mandibularis among these patients was 7.8% (25 out of 322, Table 4). Clinical oral examinations found that the great majority of mandibular tori were symmetric (84.0%), nodular (96.0%), less than 2 cm in size (96.0%), and located in the premolar area (92.0%).

Molar relationships could not be identified in 55.0% of patients due to the loss of first molars (Table 5). There was no significant difference in molar relationship between patients with and without torus mandibularis (*p* = 0.442).

Almost half (49.7%) of hemodialysis patients scored 3 on the plaque index and approximately a quarter (24.5%) scored 4 on the index, indicating poor oral hygiene among these hemodialysis patients (Table 6). However, there was no significant difference in the scoring between patients with tori and those without (*p* = 0.348).

As demonstrated by the multivariate logistic regression model, blood phosphorus level (odds ratio = 1.494, *p* = 0.029) and younger age (odds ratio = 0.954, *p* = 0.009) were major predictors for torus mandibularis (Table 7).

## 4. Discussion

A broad range of prevalence rates of torus mandibularis has been reported in the medical literature. As shown in Supplementary Table S1, the prevalence of torus mandibularis in nonuremic patients ranges from 0.9% to 58.3% [21,24,25,26,27,28,29,30,31,32,33,34,35,36,37,38]. In the uremic population, Chao et al. [18] documented a prevalence rate of 6.7% in hemodialysis patients, while Hsu et al. [39] concluded a prevalence rate of 5.3% in patients receiving peritoneal dialysis. The prevalence of torus mandibularis in the current study was 7.8%. The aforementioned studies (listed in Supplementary Table S1) were done in different patient populations and general communities, so naturally the prevalence rates varied greatly. No definitive explanation appears to account for the gap between the much higher prevalence rates found in many of these studies and the rate found in our study. In the meantime, it is worth noting that there remain limited published data on the prevalence rate of torus mandibularis specifically in the uremic patient population. The pathogenesis of torus mandibularis has long been debated and is potentially attributable to both genetic and environmental factors (including occlusal forces). Another potential contributing mechanism which has been proposed is biomechanical forces—particularly in the oral cavity—combined with cortical bone loss and trabecular expansion, as might be seen in the early stages of primary hyperparathyroidism. Further research is thus needed to confirm the underlying mechanism of torus mandibularis.

Approximately three quarters of patients in this study scored 3 or 4 on the plaque index. Oral hygiene can have a deep effect on the overall health of hemodialysis patients [40]. More than half of the patients in this study had also lost their first molars. Premature tooth loss appears to be common in patients receiving hemodialysis [41], and the likelihood of losing posterior teeth among dialysis patients can be 2.75 times greater than among their healthy peers [41]. Wilczyńska-Borawska et al. also found that nearly 70% of hemodialysis patients did not use dental prostheses and were unable to perform a satisfactory masticatory function [42].

Patients with torus mandibularis were found to be significantly younger than patients without torus mandibularis (*p* = 0.003) in this study. The pathogenic link between younger age and the formation of torus mandibularis is currently unknown. One hypothesis posits that the higher prevalence of torus mandibularis among younger patients might be attributable to nutritional and functional factors. First, the lessening capacity to chew that is more likely among older hemodialysis patients may lead to malnutrition and reduce the likelihood of torus mandibularis forming [2]. Second, with old age, tooth loss or periodontal illness may lead to a decreasing number of teeth. Over time, edentulousness results in compromised masticatory performance and in turn less occlusal stress [25].

Considering the association between age and torus mandibularis, the onset can be found as early as infancy [43]. Tori then tend to grow with age, likely resulting from constant and augmented occlusal stress from the tooth to the alveolar bone [44]. Eggen et al. [45] suggested that the formation of tori could be a dynamic process between skeletal growth and resorption. Torus mandibularis is frequently diagnosed incidentally after middle age [1,46], with its prevalence dropping after the age of 50. This reduction in prevalence might be a function of malnutrition [30,47] and a decrease in the demand of masticatory process, as well as less occlusal stress [48] due to the age-related loss of teeth. Oral tori also diminish in size after the 5th decade of life because of the decreased number of teeth following bone resorption [33].

A higher blood concentration of phosphate was found in the current study among patients with torus mandibularis than those without (*p* = 0.001). Between dialysis patients and the general population, the different prevalence rates of torus mandibularis may be related to the uremic milieu in the former group. In dialysis patients, the compromised kidney function induces phosphate retention which in turns causes hypocalcemia. The low circulating calcium level then triggers parathyroid hyperactivity, promoting phosphate excretion, decreasing calcium excretion, and increasing calcium release from bones through osteoclast activation [49]. Hyperphosphatemia also increases the concentration of circulating fibroblast growth factor 23, reduces the synthesis of active vitamin D, and produces hypocalcemia, thereby resulting in secondary hyperparathyroidism [50]. Hyperparathyroidism reduces cortical bone and enhances cancellous bone, creating changes in the skeletal structure. The loss of cortical bone and enhanced growth of trabecular bone in the oral cavity are the early indicators of uremic osteodystrophy [13]. A high prevalence of mandibular tori has also been reported in patients with primary hyperparathyroidism [13]. However, in the current study, dialysis patients with torus mandibularis showed a lower blood concentration of intact parathyroid hormone than those without tori, although the difference did not reach statistical significance (*p* = 0.636).

The great majority of the tori found in this study were symmetric (84.0%), nodular (96.0%), less than 2 cm in size (96.0%), and located in the premolar area (92.0%). Most patients were unaware of the existence of tori in their oral cavities before being enrolled to this research. Furthermore, torus mandibularis is a benign condition and the tori discovered in the current study were mostly small in size. Therefore, there was no clinical intervention initiated among these hemodialysis patients.

Our study contributed to expanding the current limited understanding of the prevalence of toris mandibularis among dialysis patients by reporting on the prevalence rate within a larger group of patients and identifying younger age and higher blood phosphorus level as potential predictors. Nevertheless, this study is limited by lacking patient recruitment from 2017 to 2020. Meanwhile, the lack of molecular and histological evaluation may limit the extrapolation of the findings of our study.

## 5. Conclusions

The prevalence rate of torus mandibularis in patients receiving hemodialysis was 7.8%. Most torus mandibularis were less than 2 cm in size, in a nodular shape, and symmetrically located in the premolar area. There was no gender difference between patients with and without torus mandibularis. Younger age and a higher blood phosphate level were significant predictors for torus mandibularis in hemodialysis patients.

## Figures and Tables

**Table 1:** The demographics, health conditions, and lifestyle factors of hemodialysis patients with and without torus mandibularis (n = 322).

Variable	All Patients (n = 322)	Patients with Torus Mandibularis (n = 25)	Patients without Torus Mandibularis (n = 297)	*p* Value
Age (Year)	61.1 ± 11.8	54.4 ± 10.4	61.6 ± 11.8	0.003 *
Sex (Female), n (%)	143 (44.4)	12 (48.0)	131 (44.1)	0.707
Hypertension, n (%)	164 (50.9)	14 (56.0)	150 (50.5)	0.598
Diabetes mellitus, n (%)	114 (35.4)	9 (36.0)	105 (35.4)	0.948
Dialysis duration (month)	107.1 ± 82.4	107.8 ± 104.1	107.1 ± 80.5	0.969
Alcohol consumption, n (%)	11 (3.4)	0 (0)	11 (3.7)	0.328
Betel nut chewing, n (%)	12 (3.7)	1 (4.0)	11 (3.7)	0.940
Cigarette habit, n (%)	28 (8.6)	1 (4.0)	27 (9.1)	0.386
Bruxism habit, n (%)	33 (10.2)	5 (2.0)	28 (9.4)	0.094

* *p* < 0.01.

**Table 2:** Laboratory data of hemodialysis patients with and without torus mandibularis (n = 322).

Variable	All Patients (n = 322)	Patients with Torus Mandibularis (n = 25)	Patients without Torus Mandibularis (n = 297)	*p* Value
Biochemistry and electrolyte				
Fasting glucose, mg/dL	117.0 ± 53.0	101.6 ± 41.5	118.2 ± 53.7	0.132
Sodium, mEq/L	137.8 ± 2.8	137.9 ± 2.5	137.8 ± 2.8	0.911
Potassium, mEq/L	4.7 ± 0.7	4.9 ± 0.6	4.7 ± 0.7	0.132
Chloride, mEq/L	99.1 ± 2.9	98.8 ± 3.2	99.1 ± 2.9	0.645
Calcium, mg/dL	9.7 ± 0.9	9.6 ± 0.7	9.7 ± 1.0	0.956
Phosphorus, mg/dL	5.0 ± 1.2	5.6 ± 1.3	5.0 ± 1.1	0.010 *
Uric acid, mg/dL	6.7 ± 1.3	6.9 ± 1.0	6.7 ± 1.3	0.604
Bicarbonate, mmol/L	21.7 ± 2.4	21.6 ± 2.2	21.7 ± 2.4	0.872
Renal function test				
Blood urea nitrogen, mg/dL	67.4 ± 17.7	71.1 ± 19.9	67.1 ± 17.5	0.274
Creatinine, mg/dL	10.3 ± 2.1	11.0 ± 2.1	10.3 ± 2.1	0.078
Estimated glomerular filtration rate, mL/min/1.73 m^2^	4.8 ± 1.1	4.4 ± 0.7	4.8 ± 1.1	0.149
Liver function test				
Aspartate aminotransferase, U/L	22.1 ± 8.9	22.7 ± 10.7	22.0 ± 8.7	0.723
Alanine aminotransferase, U/L	17.5 ± 12.9	18.8 ± 12.3	17.3 ± 13.0	0.576
Gamma-glutamyl transferase, U/L	32.2 ± 42.9	39.8 ± 71.0	31.5 ± 39.8	0.358
Alkaline phosphatase, U/L	75.0 ± 41.3	70.8 ± 22.1	75.3 ± 42.5	0.599
Lipid profile				
Total cholesterol, mg/dL	160.6 ± 33.2	159.6 ± 30.8	160.6 ± 33.4	0.886
High-density lipoprotein, mg/dL	43.2 ± 14.2	46.3 ± 15.4	42.9 ± 14.1	0.249
Low-density lipoprotein, mg/dL	106.2 ± 72.6	98.7 ± 68.	106.8 ± 73.0	0.591
Triglyceride, mg/dL	146.5 ± 117.0	148.6 ± 128.4	146.4 ± 116.3	0.927
Hemogram				
White blood cell count, 103/uL	6.4 ± 1.9	6.2 ± 2.3	6.4 ± 1.9	0.618
Red blood cell count, 106/uL	3.6 ± 0.5	3.6 ± 0.5	3.6 ± 0.6	0.730
Hemoglobin, g/dL	10.4 ± 1.2	10.4 ± 1.1	10.4 ± 1.2	0.950
Hematocrit, %	32.0 ± 3.6	32.1 ± 3.2	32.0 ± 3.6	0.889
Mean corpuscular volume, fL	89.0 ± 7.5	89.9 ± 6.2	89.0 ± 7.6	0.531
Mean corpuscular hemoglobin, pg/Cell	29.0 ± 2.8	29.2 ± 2.6	28.9 ± 2.9	0.592
Mean corpuscular hemoglobin concentration, gHb/dL	32.5 ± 1.0	32.5 ± 1.0	32.5 ± 1.0	0.843
Red blood cell distribution width, %	14.5 ± 1.4	14.1 ± 1.2	14.6 ± 1.4	0.097
Platelet count, 103/uL	184.3 ± 63.7	185.7 ± 69.2	184.2 ± 63.3	0.908
Ferrokinetics				
Iron, ug/dL	70.2 ± 53.6	62.0 ± 28.2	70.9 ± 55.2	0.426
Total iron binding capacity, ug/dL	256.9 ± 49.8	275.4 ± 51.7	255.4 ± 49.4	0.054
Ferritin, ng/mL	297.7 ± 300.4	254.7 ± 293.8	301.6 ± 301.2	0.455
Parathyroid hormone				
Intact parathyroid hormone, pg/mL	325.7 ± 311.4	297.3 ± 232.0	328.1 ± 317.3	0.636
Nutrition marker				
Albumin, g/dL	4.0 ± 0.3	3.9 ± 0.3	4.0 ± 0.3	0.656
Inflammatory marker				
High sensitivity C-reactive protein, mg/L	6.9 ± 11.1	8.1 ± 10.3	6.8 ± 11.1	0.582

* *p* < 0.05.

**Table 3:** Dialysis-related data of hemodialysis patients with and without torus mandibularis (n = 322).

Variable	All Patients (n = 322)	Patients with Torus Mandibularis (n = 25)	Patients without Torus Mandibularis (n = 297)	*p* Value
Kt/V	1.8 ± 0.4	1.8 ± 0.3	1.8 ± 0.4	0.480
Urea reduction ratio	0.8± 0.1	0.8 ± 0.1	0.8 ± 0.1	0.498
Time-averaged concentration of urea, mg/dL	39.6 ± 9.8	40.2 ± 10.5	39.5 ± 9.7	0.521
Normalized protein catabolic rate, g/kg/day	1.2 ± 0.4	1.1 ± 0.4	1.2 ± 0.4	0.701

Note: K represents dialyzer clearance of urea, t represents dialysis time, and V represents volume of distribution of urea.

**Table 4:** Clinical oral examinations of torus mandibularis in hemodialysis patients (n = 25).

Variable	n (%)
Shape	
Flat	0 (0)
Spindle	0 (0)
Nodular	24 (96.0)
Lobular	1 (4.0)
Location	
Incisor	0 (0)
Premolar	23 (92.0)
Molar	2 (8.0)
Symmetry	
Symmetric	21 (84.0)
Left	3 (12.0)
Right	1 (4.0)
Size	
>2 cm	1 (4.0)
<2 cm	24 (96.0)

**Table 5:** Molar relationships of hemodialysis patients with and without torus mandibularis (n = 322).

Category	All Patients (n = 322)	Patients with Torus Mandibularis (n = 25)	Patients without Torus Mandibularis (n = 297)	*p* Value
No first molar n (%)	177 (55.0)	13 (52)	164 (55.2)	0.442
Class I n (%)	86 (26.7)	9 (36)	77 (25.9)	
Class II n (%)	38 (11.8)	3 (12)	35 (11.8)	
Class III n (%)	21 (6.5)	0 (0)	21 (7.1)	

**Table 6:** Oral hygiene among hemodialysis patients with and without torus mandibularis based on the Turesky-Gilmore-Glickman modification of the Quigley-Hein plaque index (n = 322).

Score	All Patients (n = 322)	Patients with Torus Mandibularis (n = 25)	Patients without Torus Mandibularis (n = 297)	*p* Value
No teeth n (%)	13 (4.0)	0 (0)	13 (4.4)	0.348
1 n (%)	8 (2.5)	0 (0)	8 (2.7)	
2 n (%)	44 (13.7)	2 (8)	42 (14.1)	
3 n (%)	160 (49.7)	14 (56)	146 (49.2)	
4 n (%)	79 (24.5)	9 (36)	70 (23.6)	
5 n (%)	18 (5.6)	0 (0)	18 (6.0)	

**Table 7:** Predictors for torus mandibularis in hemodialysis patients (n = 322).

Variable	Univariate Analysis					Multivariate Analysis				
	β	Standard error	Wald test	Odds ratio	*p* value	β	Standard error	Wald test	Odds ratio	*p* value
Age (per 1 year increase)	−0.05	0.017	8.46	0.951	0.004 **	−0.047	0.018	6.881	0.954	0.009 **
Blood phosphate level (per 1 mg/dL increase)	0.46	0.181	6.426	1.584	0.011 *	0.401	0.184	4.767	1.494	0.029 *

* *p* < 0.05, ** *p* < 0.01.

## Data Availability

The datasets used and analyzed in this study are available from the corresponding author upon request.

## Supplementary Materials

The following are available online at https://www.mdpi.com/article/10.3390/ijerph18189451/s1. Table S1. Prevalence rates of torus mandibularis reported in the medical literature.
