# Supplementary material for: Torus Mandibularis in Patients Receiving Hemodialysis
Source: Int J Environ Res Public Health. 2021 Sep 7;18(18):9451. doi: 10.3390/ijerph18189451 (PMC8465652; doi:10.3390/ijerph18189451)
Supplement: Supplementary file 1 [file ijerph-18-09451-s001.zip › ijerph-1343298-supplementary (1).pdf]

Table S1. Prevalence rates of torus mandibularis reported in the medical literature.

Author(s)	Publication Year	Geographic Location	Sample Size	Patient Group	Prevalence Rate
Reichart et al [21]	1988	German	1317	Nonuremic	5.2
Reichart et al [21]	1988	Thailand	947	Nonuremic	9.2
Shah et al [25]	1992	India	1000	Nonuremic	1.4
Ruprecht et al [26]	2000	United States	1600	Nonuremic	16.9
Bruce et al [27]	2004	Ghana	926	Nonuremic	12.1
Jainkittivong et al [24]	2007	Thailand	1520	Nonuremic	32.2
Sawair et al [28]	2009	Jordan	618	Nonuremic	25.7
Simunkovic et al [29]	2011	Croatia	1679	Nonuremic	12.6
Hiremath et al [30]	2011	Malaysia	65	Nonuremic	4.6
Chiang et al [31]	2014	Taiwan	2050	Nonuremic	24.2
Yoshinaka et al [32]	2014	Japan	664	Nonuremic	29.7
Chao et al [18]	2015	Taiwan	119	Uremic (hemodialysis)	6.7
Hsu et al [39]	2016	Taiwan	134	Uremic (peritoneal dialysis)	5.3
AIZarea [33]	2016	Saudi Arabia	847	Nonuremic	9.8
Scrieciu et al [38]	2016	Romania	74	Nonuremic	9.5
Maduakor and Nwoga [34]	2017	Nigeria	292	Nonuremic	56.8
Morita et al [35]	2017	Japan	204	Nonuremic	58.3
Kumar Singh et al [36]	2017	Malaysia	2666	Nonuremic	10.5
Telang et al [37]	2019	Malaysia	4443	Nonuremic	0.9
Present study	2021	Taiwan	322	Uremic (hemodialysis)	7.8
